# Cancer of Mamma—Operation for

**Published:** 1860-09

**Authors:** T. Wilkins

**Affiliations:** Vandalia, Ill.


THE CHICAGO
### MEDICAL JOURNAL.

VOL. III. September, 1860. NO. 9.

### Original Communications.

### ARTICLE 1.

### CANCER OF MAMMA—OPERATION FOR.

BY T. WILKINS, M. D., VANDALIA, ILL.

On the 16th day of February, 1860, I was consulted in relation to a tumor of the left mamma. The individual, (a female) is 23 years of age, well formed, and intelligent; hair and eyes black, and the temperament appearing to consist of the bilious, nervous, sanguine, and lymphatic, one predominating over another in the order named. She has never conceived, and her menstrual periods for some months back have been irregular, both in the time of their appearance and in quantity. She has never had but little sickness of any kind, and no hereditary predisposition to scirrus could be traced.

In the left mamma the tumor had been discovered by her only about two or three months previous to examination. I found it to be of an oval form, about two inches in its short, and three inches in its long diameter. It moved freely, was rough and indurated, but there was no discoloration of the integument. The integument generally was of a healthy color. Her general health appeared to be moderately good, but finding most of the symptoms well marked, I did not hesitate to pronounce the disease scirrus.

*Treatment.*—This was both external and internal. The external consisted of ground slippery-elm poultices, camphor dissolved in olive oil, camphoreted mercurial ointment, tinct. of iodine, iodine ointment, a solution of gold, &c., &c. An ointment made of spruce pine was suggested by the patient and her husband, and, as I had no objections, it was used also. This appeared to allay pain to a considerable extent, and for that purpose, at least, might be tried again.

The principal remedies used internally were mild cathartics, alterants, tonics, and sedatives; some of which were iodine, iodide of potassa, hyd. cum. creta, pulv. doveri, opium, hyoscyamus, quinine, tinct. cinchonia, and occasionally diffusible stimulants. The corrosive chloride of mercury was used in alterative doses, till its constitutional effect was perceived. This appeared to hold the growth of the tumor in check more than any remedy I used, and is worthy, I think, of some farther trial. I used also some of the preparations of iron, particularly the lactate (which was recommended by Prof. Brainard,) and, although to it I attribute the *cure* of one case of cancer in which I used this article some years since, in *this* case it appeared to do but little, if any good; the article I used, however, I did not consider good.

In the case alluded to above, of cure, I might possibly have been mistaken as to the nature of the disease. But to digress a little—let me say that there is not half the attention paid to the treatment of scirrus which should be, for the reason that most of the writers and teachers pronounce it incurable. As long as this is the case there will be no improvement made in the treatment of this formidable disease. Ague was once thought to be incurable.

To return: The tumor on the whole continued gradually to increase. Lancinating and pungent pains were often felt, which would occasionally shoot up into the axilla, and one of the axillary glands became involved. The mammary tumor grew in every direction, till nearly the whole gland was involved, and it was close to the integument but was not quite adherent to it.

I advised extirpation with the knife from the beginning of the treatment, but it was some time before I could get the consent of my patient. After this was obtained I lost but little time, and on the 14th of April the operation was performed. An incision was extended into the axilla and the indurated axillary gland removed. But little blood was lost, and no pain experienced, the patient having been placed completely under the influence of chloroform. Considerable emesis occurred the evening and night following the operation, which was relieved by opium, brandy, and mustard to the epigastric region. It was caused, I think, by the chloroform. The shock to the system was inconsiderable.

April 16th—I visited the patient, opened and dressed the wound. Considerable suppuration had occurred, but this diminished gradually at nearly every dressing. For some time it was dressed every other day, and it healed rapidly. Two or three suspicious looking spots were well cauterized with caustic potassa, and the wound was kept from healing so rapidly by the occasional use of this article. A little while after its application it was neutralized with diluted acetic acid. The granulations, a part of the time during the treatment, were unhealthy, which condition, however, was corrected by the use of lunar caustic. During the after treatment such articles were used as appeared to be indicated, more especially tonics, stimulants, and alterants. She is now, and has been for more than a week, using sarsaparilla and iodide of potassa.

Yesterday (May 23d) I saw her. She prsents the appearance of general good health, the wound is consolidated, filled with granulations, and nearly healed up. It healed first in the axilla. Perhaps it would not be uninteresting to state that the second time of the appearances of the menses occurred a little over four weeks after the operation. The first time immediately (within a day or two) after the operation, the second just four weeks after the first.

During the whole time the diet has been nutritious, most of the time the appetite has been good, exercise has been allowed according to ability, both in and out of doors. Yesterday she came to town, the distance being about eight miles, and the case at this time promises to be successful.

The idea of keeping the wound from healing immediately after the extirpation of a cancer is not original with me, but I think it may have a tendency to insure the cure.

July 26th, 1860—The wound has been healed up for several weeks, and the individual looks hearty and robust.

